# Population ecology and the management of whale watching operations on a data‐deficient dolphin population

**DOI:** 10.1002/ece3.5565

**Published:** 2019-08-22

**Authors:** Maddalena Fumagalli, Amina Cesario, Marina Costa, Giuseppe Notarbartolo di Sciara, John Harraway, Elisabeth Slooten

**Affiliations:** ^1^ Department of Zoology University of Otago Dunedin New Zealand; ^2^ Tethys Research Institute Milano Italy; ^3^ The Swire Institute of Marine Science University of Hong Kong Hong Kong SAR China; ^4^ South Atlantic Environmental Research Institute (SAERI) Stanley Falkland Islands; ^5^ Department of Mathematics and Statistics University of Otago Dunedin New Zealand

**Keywords:** CJS models, inequality model, lagged identification rate, Red Sea, spinner dolphin, tourism management, whale watching

## Abstract

1. Whale watching is a popular commercial activity, producing socio‐ecological benefits but also potential long‐term effects on the targeted cetacean population. This industry is currently developing in data‐deficient contexts in a largely unregulated fashion. Management schemes should adopt precaution and be informed by the relevant literature, but would be more effective if the assessment of the target population vulnerability, biological impacts, and management implications was drawn from site‐specific data.
2. This paper focuses on a reef‐associated, data‐deficient population of spinner dolphins in the Egyptian Red Sea. In Satayah Reef, new information on population size and dynamic parameters were documented using visual observation and photo‐identification‐based capture–recapture methods (Cormack–Jolly–Seber time‐since‐marking model).
3. Dolphins occurred on 98% of the survey days. Average school size was 66 individuals (±42.1 *SE*), with most groups including calves. The population was equally divided into recurrent and transient individuals. An “emigration + mortality” model best described residence at the site. Five recurrent males (5% of the Satayah population) provided connectivity between this and the geographically close population of Samadai Reef.
4. Average annual survival probability was 0.83 (±0.06 *SE*) in the year following first capture and 0.99 (±0.06 *SE*) for recurrent individuals. Mean yearly population sizes ranged 143–207 individuals.
5. The study had the power to detect a 30% decline in the population, but not the rate of change in abundance estimated from the data (*r* = 0.018 ± 0.04), which would have required a 3‐ to 5‐times longer study.
6. *Synthesis and application*: These findings advance the assessment of the Satayah population's intrinsic vulnerability and have three major management applications: (a) the delineation of management units; (b) the identification of key indicators for future impact monitoring and assessment; and (c) realistic estimates of the statistical power for trend detection. Based on our results, we recommend supporting future research, devising site‐specific time–area closure plans, and integrating them in a regional scheme. Approaches employed in this case study can inform the management of whale watching industries targeting other data‐deficient populations.

## Graphical abstract

As whale watching develops at new destinations and affects cetacean units still poorly known, investigating the key features of wild populations allows a preliminary assessment of their vulnerability and can inform management for their conservation. This case study applies this approach on an Egyptian spinner dolphin population targeted by unregulated tourism in its resting area. Findings on the occurrence of sensitive segments, individual long‐term fidelity to the resting site, and relatively small population size suggested high vulnerability; hence, an urgent, precautionary site‐specific management intervention was recommended.

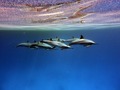

## 1 INTRODUCTION

Whale watching (WW), or the viewing of free‐ranging cetacean in the wild (Parsons et al., 2006), is a popular activity worldwide (Hoyt, 2018). WW operations have associated socio‐ecological benefits (Corkeron, 2004; Curtin, 2009; Orams, Forestell, & Spring, 2014), but can detrimentally affect the behavior of the target animals (Christiansen, Lusseau, Stensland, & Berggren, 2010; Fumagalli et al., 2018; Lundquist, Gemmell, & Würsig, 2012; Lusseau, 2003; Stockin et al., 2008) and can lead to long‐term population‐level effects, including displacement and decline (Bejder et al., 2006; Lusseau & Bejder, 2007). International organizations concerned with WW are now taking a precautionary stance and urging the adoption of appropriate mitigation measures (Convention on Migratory Species, 2017), while encouraging research on robust predictive models (e.g., LaWE, International Whaling Commission, 2009; MAWI, International Whaling Commission, 2018). WW is anticipated to grow further, especially in developing countries (Cisneros‐Montemayor, Sumaila, Kaschner, & Pauly, 2010), including contexts that are likely data‐deficient and poorly regulated. In these conditions, managing the risk of potential impacts requires the adoption of a precautionary principle and relying on the relevant literature (Bejder et al., 2006). Approaches to control the industry have so far included the implementation of code of conducts and guidelines (e.g., Zanzibar; Christiansen et al., 2010), certification schemes (e.g., self‐regulated cooperatives in Lovina, Bali; Mustika et al., 2012), land‐based watching (e.g., Fernando de Norohña, Brazil; Carli, Silva, & Silva, 2017), and/or time–area closures (e.g., Samadai Reef in Egypt; Notarbartolo di Sciara et al., 2009), either on voluntary or mandatory bases, among others.

In order to manage WW operations effectively, site‐specific information on the vulnerability of individuals and populations targeted are needed (Higham, Bejder, & Lusseau, 2009). The importance of such information is trifold. In a developing WW scenario, site‐specific information allows the assessment of the vulnerability of the targeted cetacean population or subpopulation. Indicators of vulnerability include biological and ecological conditions that regulate individual exposure, sensitivity, and recovery to human interactions (De Lange, Sala, Vighi, & Faber, 2010; De Lange, Van der Pol, Lahr, & Faber, 2005), including age, sex and reproductive classes, body condition, behavior, frequency of exposure to interactions (Christiansen & Lusseau, 2014), and other species‐ or site‐specific features. A combination of observational and photo‐identification‐based capture–recapture (CR) studies can provide such information (Cribb, Miller, & Seuront, 2012; Karczmarski et al., 2005; Norris et al., 1994; Parra, Corkeron, & Marsh, 2006). Individual cetaceans are often recognized from the marks that naturally accumulate on or near the dorsal fin, and their occurrence in the study area is recorded by means of photo‐identification (photoID), a commonly used technique to collect photographic evidence of the individuals encountered (Hammond, Mizroch, & Donovan, 1990). The capture histories of distinctive individuals (i.e., vectors of their presence and absence at sampling occasions) are analyzed in CR models to estimate individual site fidelity and population parameters (Hammond, Mizroch, & Donovan, 1990; Kendall, Pollock, & Brownie, 1995; Otis et al., 1978; Pollock, 2000; Seber, 1982). Among these parameters, residence, female reproductive rate, individual survival, and population size have been proposed as valid metrics to assess the biological impacts of WW activities (Bejder et al., 2006; Lusseau, Slooten, & Currey, 2006). Population ecology can also help monitor the efficacy of implemented measures in safeguarding wild populations (Gormley et al., 2012). Finally, site‐specific studies can support management and decision‐making processes through the identification of targets of protection (De Lange et al., 2010), diagnostic indicators for adaptive management (e.g., Limits of Acceptable Change (LAC); Stankey et al., 1985; Duffus & Dearden, 1990; Higham, Bejder, & Lusseau, 2009), and considerations on effective study designs (e.g., Gerrodette's inequality model; Gerrodette, 1987).

**Figure 1:**
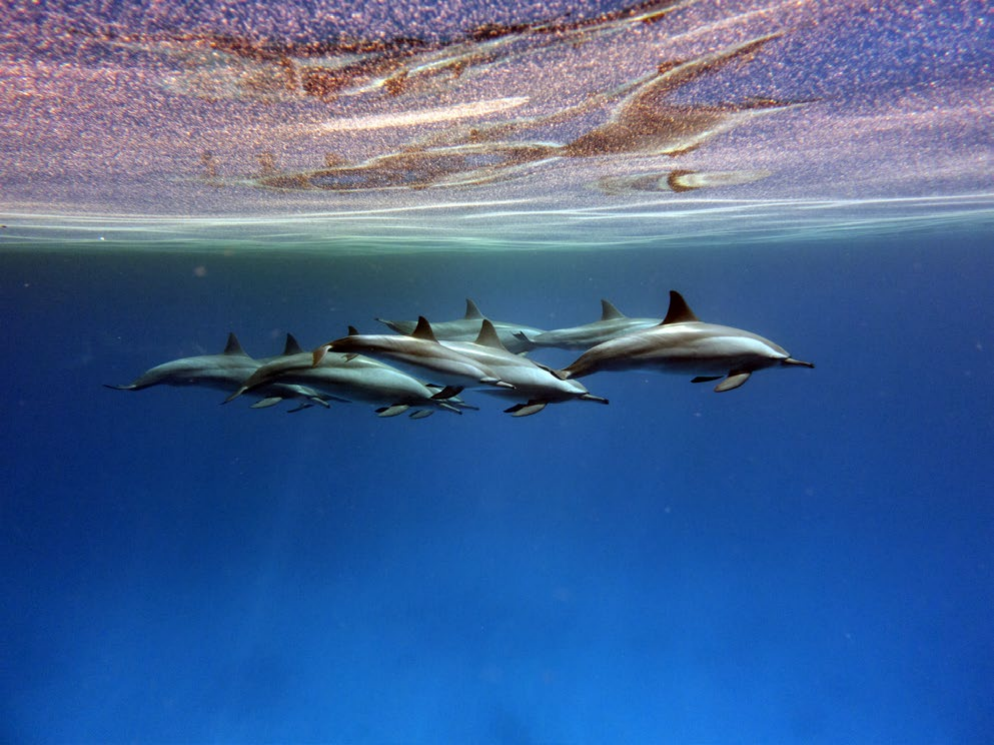
A group of spinner dolphins in a resting area off the Egyptian coast (Photo by A.Cesario/HEPCA)

This research presents a case study on a reef‐associated population of spinner dolphin (*Stenella longirostris*) in the Egyptian Red Sea (Figure 1). This species is particularly vulnerable to WW activities (Johnston, 2014; Tyne et al., 2017) because of its exposure in critical resting areas (Norris et al., 1994), sensitivity to associated disturbances (Courbis & Timmel, 2009; Fumagalli et al., 2018; Heenehan et al., 2017; Lammers, 2004; Timmel et al., 2008), and lack of resilience to disruptions (Tyne et al., 2017). In the Egyptian Red Sea, the rapid growth of a commercial WW industry at Samadai Reef in the early 2000s (O'Connor et al., 2009) generated serious concern among the local stakeholders, resulting in the prompt implementation of a precautionary, site‐specific management plan (Notarbartolo di Sciara et al., 2009), and dedicated research efforts (Cesario, 2017; De Montpellier, 2007; Fumagalli, 2016; Fumagalli et al., 2018; Notarbartolo di Sciara et al., 2009; Ponnampalam, 2005; Shawky & Afifi, 2008; Shawky et al., 2015). The management plan in Samadai Reef substantially reduces behavioral disruptions caused by human interactions, which are instead documented as pervasive and severe at the nonmanaged resting area at Satayah Reef (Fumagalli et al., 2018). There, in 2009, tourism was reported as “opportunistic” with a potential for further development (O'Connor et al., 2009). Indeed, as of 2014, 90+ swimmers and 10 inflatable boats could simultaneously approach a resting school during dedicated swim‐with activities, and the active, invasive interactions could last for up to 9 hr daily (Fumagalli, 2016). WW operations at Satayah Reef have been unregulated and unrestricted since inception, in the mid‐2000s (O'Connor et al., 2009). The paucity of information on the spinner dolphins using Satayah Reef limits the ability to gauge their vulnerability, detect biological impacts, and inform management of WW activities at this site. To address this limitation, we report original information on resting school demographic composition, individual site fidelity, and population size in 2006 and 2010–2013. This case study explores and reflects on the role of site‐specific population ecology in managing emerging WW activities and its implications for conservation. Set in a data‐poor scenario, we argue that our experience can guide and inspire efforts in similar contexts, where the WW industry may expand uninformed.

## 2 MATERIALS AND METHODS

**Figure 2:**
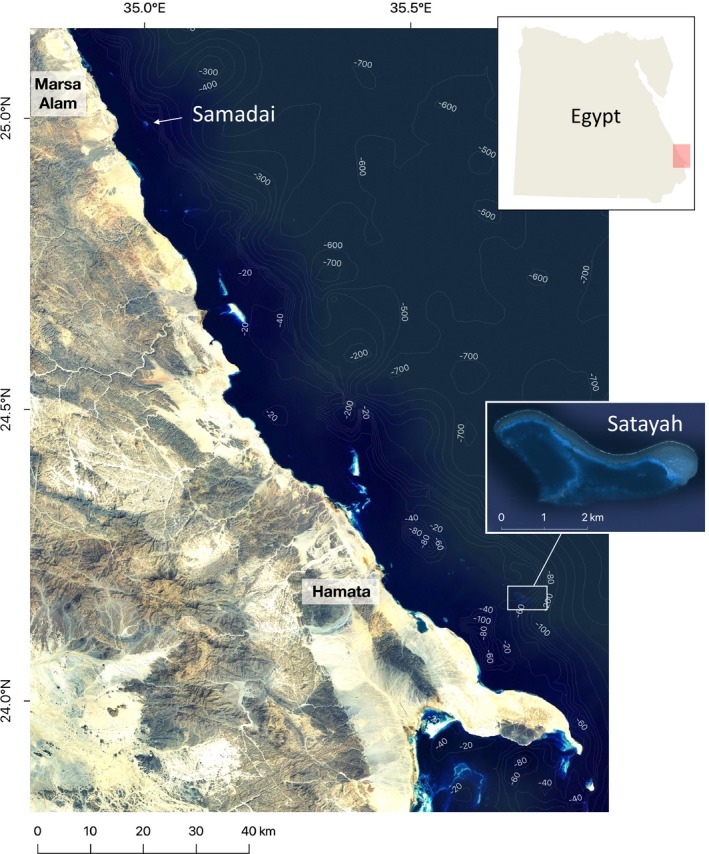
Location of Satayah Reef in the Southern Egyptian Red Sea. The map was created using ESRI's World Imagery in QGIS3 (QGIS Development Team, 2018). The aerial image of Satayah Reef was obtained from Google Maps Satellite

Satayah Reef (24.16°N, 35.70°E) is located 30 km southeast of Hamata, north of the Ras Banas peninsula, and 120 km south of Samadai Reef (24.99°N, 35.00°E) (Figure 2). The reef is composed of two lagoons, each extending approximately 1.4 km^2^. Surveys at the site took place on 53 days in 2006 and 2010–2013, as indicated in Table 1.

**Table 1:** Summary of the sampling effort per year: survey dates (Jun = June, Jul = July, Aug = August), sampling effort, number of encounters, number of photoID sessions matching the criterion for inclusion in the analyses (highlighted in bold in “Survey dates”) and number of distinctive individuals identified each year

Year	Survey dates	Sampling effort (days)	No. Encounters	No. valid PhotoID occasions (total occasions)
2006	Jul: **22–24** Aug: **20**	4	4	4^a^
2010	Jun: 9, **10**, **12**, **14**, **15**, 23, **25**, **26**; Aug: 2, 10, 11, 18, **20**, 21	14	14	7 (13)
2011	Jul: **23, 24, 25, 26, 27, 28, 29**, 30	8	8	7 (7)
2012	Jun: **13**, **14**, **18**, 20, 25**, 26, 27, 28**, 29; Jul: 9, **18**, 19	12	11	7 (9)
2013	Jul: **17**, 18, **19**, **20**, 21, 22, 23, 24, **25**, 26, **27**, **28**, **29**, **30**, **31**	15	15	9 (15)

a As described in methods, validity criteria were relaxed for the 2006 sessions.

Visual observations to detect the presence of dolphins were carried out from a dedicated, stationary vessels moored in the western lagoon of Satayah Reef. Observations started at dawn, or at arrival on site, and ended at sunset, or when research efforts were interrupted for logistical reasons. The sighting of the first dolphin in the lagoon from the stationary vessel marked the beginning of an “encounter,” which ended with the departure of the last dolphin, or with the end of the daily observations. Given the structure of the reef protecting the lagoon from the mainly northerly winds, all surveys were conducted in calm sea conditions (Beaufort sea state <2) even on high wind days.

### 2.1 School composition

School size (total number of individuals in the lagoon) and composition in age classes were estimated in 2010–2013 during 35 photographic sessions (see below). An individual was considered “calf” if <¾ the size of an adult and in regular association with an adult, or “newborn calf” if it showed obvious fetal folds (Norris et al., 1994). All other individuals were “adult.” The occurrence and number of females in early or advanced pregnancy stage (see in Appendix) were assessed visually during underwater sessions in 2011–2013. Independent field estimates provided by experienced researchers were averaged to estimate the mean school size, and the number of calves and newborn calves in a school.

### 2.2 Photographic identification

When dolphins were detected in the lagoon of Satayah Reef, a first photographic session was carried out for photoID purposes. If the photographers deemed the coverage of the school insufficient, or when the encounter extended throughout the day, at least one more photographic session was performed (ideally one in the morning and one in the afternoon) in order to increase opportunities to cover the entire school and to account for possible changes in the daily school composition. The duration and number of sessions were also context‐dependent: when the co‐occurrence of tourism activities made it difficult to maneuver around the dolphin school, causing concerns over the quality of data collected as well as the welfare of the animals, sessions were interrupted and resumed at a later stage. Photographic sessions did not follow pre‐established line transect and were aimed to provide even coverage of all individuals in each group found in the lagoon. Sessions were conducted from the surface, on board 4‐ to 6‐m inflatable boats equipped with 45–150 HP outboard engines, and/or underwater, snorkeling in proximity to the dolphins. Underwater photoID was shown to provide good coverage of the dolphin group (0.84 ± 0.15 *SD*; Cesario, 2017) and deliver information not available from the surface, thus was preferred over boat‐based photoID when conditions allowed. In both cases, photographers attempted to equally sample all individuals and groups in the lagoon, irrespective of their distinctiveness, behavior, sex and age, and followed a code of conduct to minimize disturbance to the school (details in Appendix).

All sessions carried out in the same day, hence on the same encounter, were pooled together in a photographic occasion. To promote consistent and higher quality assessment of individual presence (see Ottensmeyer & Whitehead, 2003), only 30 occasions with a number of photographs at least three times the estimated school size were retained for further analyses (7 in 2010, 7 in 2011, 7 in 2012, and 9 in 2013). In addition, four occasions from 2006 were included in the creation of the catalogue of individuals and in the assessment of individual site fidelity to provide historical perspective.

PhotoID images were assessed by experienced researchers for photographic quality and individual distinctiveness using protocols modified from the literature (Friday, Smith, Stevick, & Allen, 2000; Urian et al., 2015) and consistent with studies on the Samadai population (Cesario, 2017; see Appendix). Very Distinctive (D1), Distinctive (D2), and Marked (D3) noncalf individuals in photographs of Excellent and Very Good quality were assigned unique codes and added to the Satayah catalogue (definition of distinctiveness and photographic quality categories in Appendix). Photographic evidence of sex‐specific features allowed sex determination of males (genital area; extruded penis or postanal hump) and females (genital area; pregnancy; prolonged association with a calf). Catalogued individuals were ranked as Recurrent (encounters in at least two years) and Transient (multiple encounters in one year; including True Transients, encountered only once; Pradel et al., 1997) based on the capture history. Data processing was software‐assisted with Discovery (Gailey & Karczmarski, 2012).

### 2.3 Site fidelity

The lagged identification rate (LIR) was estimated in SOCPROG 2.7 (Whitehead, 2015) to test scenarios in which there is no change in the individuals (closed model), individuals leave and never return (emigration + mortality), leave and return (emigration + reimmigration), or a combination of the last two (emigration + reimmigration +mortality) (Whitehead, 2001). Model selection was based on the lowest quasi‐likelihood Akaike information criterion (QAIC) (Whitehead, 2007). Supported models fell within 2 units (Burnham & Anderson, 2002). Confidence interval and standard error of parameter estimates were calculated using nonparametric bootstrap techniques (100 replicates) (Whitehead, 2007).

### 2.4 Connectivity

The Satayah and Samadai catalogues were compared to assess the presence of common distinctive individuals in the two populations. The Samadai catalogue of 203 individuals included photographic material collected over 198 encounters in 2006 and 2010–2014.

### 2.5 Population parameters

The capture histories of Highly Marked Individuals (HMIs, including D1 and D2) in 2010–2013 were pooled in four yearly occasions (2010, 2011, 2012, and 2013) to estimate annual survival and capture probabilities, and population size in program MARK (White & Burnham, 1999). The Global test on the dataset showed overdispersion (*χ* ^2^ = 6.994 and *p* = .14), and CR strict assumptions on capture and survival heterogeneity (due to, among others, transience; see Appendix) were tested in UCare (Choquet, Reboulet, Pradel, Gimenez, & Lebreton, 2002). Preliminary analysis of the individual capture histories anticipated the occurrence of transients in the sample. In order to minimize biases on apparent survival (Pradel et al., 1997) and abundance (Pollock et al., 1990), we employed Cormack–Jolly–Seber time‐since‐marking (TSM) models for yearly abundance estimates.

TSM models estimate survival for the year following first capture (M1) and the subsequent years (M2) (Brownie & Robson, 1983; Pradel et al., 1997), thus quantifying survival over the first interval after capture, when both recurrents and transients are in the sample, and for successive years, hence representing only recurrent individuals.

TSM models can be used to model survival as constant, time‐since‐marking and year‐dependent. Candidate TSM models in this study included therefore combinations of constant (.), time‐since‐marking (*t*), and year‐dependent (*y*) survival (*φ*) for the year after the first capture (M1) and for successive year (M2), and constant (.) and year‐dependent (*t*) capture probabilities (*p*). The best model minimized the small‐sample Akaike's information criterion (AICc, Hurvich & Tsai, 1989). A Horvitz–Thompson type estimator was used to estimate the total number of HMIs in the population at occasion *i* (*N*_HMIi_), its standard error, and 95% confidence intervals (Loery, Nichols, & Hines, 1997; McDonald & Amstrup, 2001) (Formulae in Appendix).

The Mark Rate (*θ*), or the proportion of HMIs in this population, was estimated in Fumagalli (2016) as the number of HMIs over the total number of individuals portrayed in a subset of 800 randomly chosen pictures of Excellent and Very Good quality. It was assumed constant over time and used to scale the estimated number of HMIs (*N*_HMIi_) to yield total population size estimate (*N*), its standard error (*SE_N_*), and log‐normal confidence interval (95CI*_N_*) (Burnham, Anderson, White, Brownie, & Pollock, 1987; Williams, Nichols, & Conroy, 2002) (Formulae in Appendix).

### 2.6 Power analyses for population trends

The simplified equation of Gerrodette's inequality model (Gerrodette, 1987), *r* ^2^ *n* ^3^ ≥ 12CV^2^(*Z_α_*_/2_ + *Z_β_*)^2^, combines information on population rate of change (*r*), number of estimates available (*n*), coefficient of variation (CV), and probabilities of Type I (*Z_α_*_/2,_ one‐tailed) and Type II (*Z_β_*) errors, to calculate how large a trend could have been detected with the data available, and how long a survey would have been required to detect the observed trend. Error probabilities were set to .05 for a 95% power to detect a change (Gerrodette, 1987; Parra, Corkeron, & Marsh, 2006). The probability of making a Type II error (*β*) was set also to .20 for a more conservative 80% power (Tyne et al., 2016). The overall fractional change in population size and the annual rate of change were calculated with formulae in Gerrodette (1987; Appendix) assuming a uniform exponential trend.

## 3 RESULTS

### 3.1 School composition

Dolphins were sighted on 52 of the 53 days spent on site. Satayah schools encountered in June–July 2010–2013 averaged 66 individuals (±42.1 *SE*, range: 6–180, *n* = 35), of which three were calves (3.4 ± 2.12 *SE*) and two newborn calves (1.8 ± 2.17 *SE*). Only one encounter featured exclusively adult individuals. The presence of pregnant females was assessed during 22 encounters and ranged from 0 to 11 individuals, with an average of 2.6 (±0.54 *SE*) pregnant females per school.

### 3.2 Photographic identification

Table 1 provides a summary of the photographic effort over the study period. A total of 14,184 images were scored for quality and distinctiveness of the individual fins portrayed. The Satayah catalogue included 106 individuals encountered on average five times (±4 *SD*, range: 1–17) over 34 occasions between 2006 and 2013. Approximately half of them were first encountered in 2006. A total of 56 individuals were Recurrent and 50 Transients, of which 26 were True Transient (Table 2). Most Recurrent individuals were males; Transients were mainly of unknown sex (Table 2).

**Table 2:** Composition of the Satayah catalogue of distinctive individuals in sex and occurrence categories (Recurrent = encountered in 2+ years; Transient = encountered multiple times in one year; True Transient = encountered once)

	Male	Female	Unknown	Total
Recurrent	38	9	9	56
Transient	8	0	16	24
True transient	11	0	15	26
Total	57	9	40	106

### 3.3 Site fidelity

**Figure 3:**
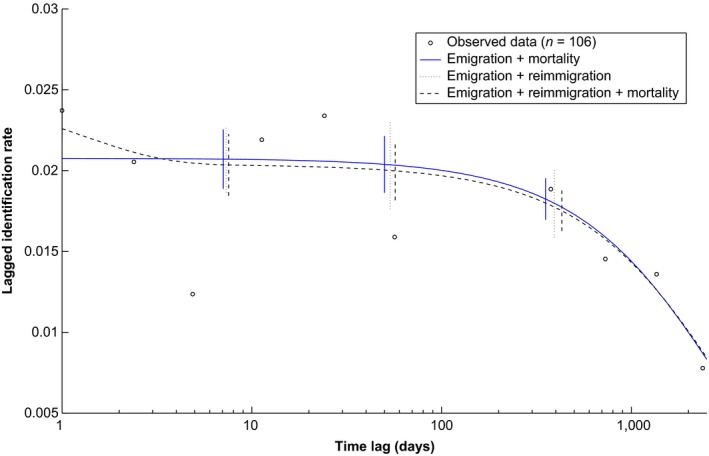
Observed and modeled lagged identification rate over time lag of Highly Marked Individuals encountered at Satayah Reef in 2006–2013. Bars show bootstrap‐estimated standard errors (100 permutations)

The best model “emigration + mortality” predicts 42–58 individuals in Satayah Reef at any given time during the study period (2006, 2010–2013), with mean residence times of 2,736 days (approx. 7 years) (Table 3). The lagged identification rate did not level off above zero at longer time lags, hence excluding residence and/or reimmigration in the site (Whitehead, 2001) (Figure 3). The supported “emigration + reimmigration” model was therefore rejected.

**Table 3:** Residency parameters (±*SE*) and bootstrapped 95% confidence intervals for distinctive individuals encountered in 2006 and 2010–2013 at Satayah Reef. Best fitting model in bold

Model	QAIC	ΔQAIC
Closed	15,326.18	109.96
*N*	66 ± 3.3 (59–72)	
Emigration + mortality	**15,216.22**	**0**
*N*	48 ± 4.2 (42–58)	
*a*	2,736 ± 703 (1,974–5,006)	
Emigration + reimmigration	15,218.22	2
*N*	48 ± 4.3 (40–55)	
*a*	2,736 ± 1,238 (55–3,949)	
*b*	1.15 E + 14 ± 1.8 E + 14 (38–6.5 E + 14)	
Emigration + reimmigration +mortality	15,218.78	2.56
*N*	39 ± 6.8 (15–51)	
*a*	6.9 ± 8,057,476.3 (0–1,813)	
*b*	1.6 ± 5.1 E + 6 (0–1,179)	
*δ*	0.0003 ± 8.9E−05 (0.001–0.0005)	

Abbreviations: a, mean residence time (days) in Satayah Reef; b, mean residence time (days) outside Satayah Reef; *N*, mean population in Satayah Reef at any given time; δ, rate of mortality or permanent emigration (notation follows (Whitehead, 2001).

### 3.4 Connectivity

Five distinctive individuals appeared in both Samadai and Satayah catalogues of 203 and 106 individuals, respectively. These were all males, Recurrent at Satayah Reef, where they were first encountered in 2006, and True Transient at Samadai Reef (one encountered in January 2006, four in the same occasion in February 2012).

### 3.5 Population parameters

Tests on CR assumptions on the capture histories of 84 HMIs indicated no sign of short‐term trap dependence (TEST2.CT: *N *= −1.7431, *p*_two‐sided_ = .08), and confirmed the transience signal in the sample (TEST3.SR: *N* = 1.9034, *p*_one‐sided_ = .028). The goodness‐of‐fit test on the TSM models showed underdispersion (median c‐hat < 1), and c‐hat was conservatively set to 1. The best TSM model (Model no.1 in Table 4) predicts that the probability of surviving and remaining in the study area is higher for individuals previously encountered. Apparent survival probability increased from 83% in the year following first capture (*φ*_M1_ = 0.83 ± 0.06 *SE*), when both transients and recurrent individuals are in the sample, to 98% in successive years (*φ*_M2_ = 0.98 ± 0.05 *SE*). Capture probability was constant at .68 (*SE* = 0.05) (“Model no. 1,” Table 4). *φ*_M1_ and *φ*_M2_ weighted averages (across the best and the two competitive models) were 0.83 (*±*0.06 *SE*) and 0.99 (*±*0.06 *SE*), respectively. The competitive models were rejected on the basis of the nonsignificant results of the likelihood‐ratio test (*χ* ^2^ = 0.125, *df* = 1, *p* = .72 for Model 1 and 2, and *χ* ^2^ = 0.01, *df* = 1, *p* = .97 for Model 1 and 3).

**Table 4:** TSM model selection for the Satayah population

No.	Model	ΔAICc	AICc weight	Model likelihood	No. of parameters
1	***φ*_TSM_(./.)*p*(.)**	**0.00**	**0.41**	**1**	**3**
2	*φ*_TSM_(./*t*)*p*(.)	1.99	0.15	0.37	4
3	*φ*_TSM_(./*y*)*p*(.)	2.12	0.14	0.34	4
4	*φ*_TSM_(*y*/.)*p*(.)	2.98	0.09	0.22	5
5	*φ*_TSM_(./.)*p*(*y*)	4.02	0.06	0.13	5
6	*φ*_TSM_(*y*/*t*)*p*(.)	4.99	0.03	0.08	6
7	*φ*_TSM_(*y*/*y*)*p*(.)	5.16	0.03	0.08	6
8	*φ*_TSM_(./*t*)*p*(*y*)	6.13	0.02	0.05	6
9	*φ*_TSM_(./*y*)*p*(*y*)	6.14	0.02	0.05	6
10	*φ*_TSM_(*y*/.)*p*(*y*)	6.86	0.01	0.03	7
11	*φ*_TSM_(*y*/*y*)*p*(*y*)	6.86	0.01	0.03	7
12	*φ*_TSM_(*y*/*t*)*p*(*y*)	6.97	0.01	0.03	8

Abbreviations: (.) = constant; *p*, capture probability; pi, capture probability at occasion i; *t*, time‐since‐marking; *y*, year‐dependent parameter; ΔAICc, Difference in AICc with the best model (in bold); *φ*, survival; *φ*_TSM(M1/M2)_, survival under TSM model after first (M1) and successive captures (M2).

The Horvitz–Thompson type estimator adjusted with the Mark Rate (*θ* = 0.39 ± 0.018 *SE*; Fumagalli, 2016) returned yearly total population sizes ranging between 143 and 207 individuals (Table 5).

**Table 5:** Estimates of Highly Marked Individual population size (*N*_HMIi_) and total population size (*N_i_*) at occasion *i* based on 2010–2013 capture histories

Model	Year	Details	*N*_HMIi _(*SE*_HMIi_)	95CI_HMIi_	*N_i_* (*SE_Ni_*)	95CI*_Ni_*
TSM		*φ*_TSM_ (./.)*p*(.) *p* = .68, var(*p*)=.0027				
	2010	*n*_1_ = 55	81 (6.2)	69–93	207 (15.8)	178–241
	2011	*n*_2_ = 38	56 (4.3)	47–64	143 (10.9)	123–166
	2012	*n*_3_ = 44	65 (4.9)	55–74	166 (12.7)	143–193
	2013	*n*_4_ = 52	76 (5.8)	65–88	196 (15.0)	169–228

Abbreviations: 95CI_HMIi_, 95% confidence interval of *N*_HMIi_; 95CI*_N_*, 95% confidence interval of *N*; *N*_HMIi_, number of Highly Marked Individuals; *n_i_*, HMIs at occasion *i*; *N_i_*, number of individuals; *p*, capture probability; *SE*_HMIi_, standard error of *N*_HMIi_; *SE_N_*, standard error of *N*; var(*p*), variance of *p*; *φ*_TSM_, survival under TSM model.

### 3.6 Power analyses for population trends

The study had a high power (1 − *β* = .95) to detect a constant rate of change as little as 0.13 per year, which would have resulted in a 34% population decline or 44% increase over the course of the study (Table 6). A smaller 0.10 rate of change, resulting in a 27% decline or 33% increase in the population, would have been detected with 80% power. The observed rate of change, calculated from the population size annual estimates, was much smaller (*r* = 0.018 ± 0.04 *SE*) and would have gone unnoticed in the present study. Detecting such a change with 95% power would require 15 years of similar annual surveys or 12 years with 80% power. By that point, approximately a fifth of the population would have been lost (Table 6).

**Table 6:** Annual rates of population change and number of surveys required to detect trends in population size

Annual rate of change (*r*)	95% power				80% power			
	Number of surveys required (*n*)	Number of years to detection [*t*(*n*−1)]	Total % change at detection for decreasing population [(1−*r*)^(^ *^t^* ^(^ *^n^* ^−1)^−1]	Total % change at detection for increasing population [(1 + *r*)*^t^* ^(^ *^n^* ^−1)^−1]	Number of surveys required (*n*)	Number of years to detection [*t*(*n*−1)]	Total % change at detection for decreasing population [(1−*r*)*^t^* ^(^ *^n^* ^−1)^−1]	Total % change at detection for increasing population [(1 + *r*)*^t^* ^(^ *^n^* ^−1)^−1]
.01	22	21	−19	23	19	18	−17	20
.02	14	13	−23	29	12	11	−20	24
.03	10	9	−24	30	9	8	−22	27
.04	9	8	−28	37	8	7	−25	32
.05	8	7	−30	41	7	6	−26	34
.06	7	6	−31	42	6	5	−27	34
.07	6	5	−30	40	5	4	−25	31
.08	6	5	−34	47	5	4	−28	36
.09	5	4	−31	41	5	4	−31	41
.10	5	4	−34	46	4	3	−27	33
.11	5	4	−37	52	4	3	−30	37
.12	5	4	−40	57	4	3	−32	40
.13	4	3	−34	44	4	3	−34	44
.14	4	3	−36	48	4	3	−36	48
.15	4	3	−39	52	3	2	−28	32

Note

Based on Gerrodette's inequality model (1987), with 95% and 80% power, yearly survey intervals (*t* = 1) and constant coefficient of variation (CV = 0.08).

## 4 DISCUSSION

The investigation of the Satayah spinner dolphin population provided an opportunity to reflect on the value of simple but site‐specific information at individual and population level in complementing the knowledge derived from the literature to better understand and manage the risk of WW impacts on wild populations in a data‐poor and unregulated context.

In June and July, spinner dolphins regularly occurred at Satayah Reef in schools of 66 individuals mixed in age, sex, and reproductive classes, including pregnant females. Approximately half of the 106 Highly Marked individuals in the Satayah catalogue were first recorded during a 4‐day pilot survey carried out in 2006. The population was found equally divided in a group of recurrent individuals, repeatedly encountered over the study period, and a group displaying transient patterns in the site. A model of emigration and mortality best predicted the individual site fidelity. There was some connectivity between Satayah and Samadai reefs, with five males (5% of the Satayah and 2% of the Samadai distinctive individuals) encountered in both locations. The survival probability was high for recurrent individuals and yearly population sizes ranged 143–207 individuals under TSM models. Assuming that a trend in population size as the one estimated had indeed occurred, the study would have failed to detect it. The analysis shows that detection of such trend would occur only after 12 or 15 similar yearly surveys (with 80% and 95% power, respectively). As timely detection of a negative trend is particularly important for small, isolated units (Thompson et al., 2000; Wilson, Hammond, & Thompson, 1999), solutions to enhance the likelihood of detection through greater precision of the estimates and/or increased sample size should be considered.

The mixed composition and size estimates of Satayah schools fell in ranges reported for spinner dolphins elsewhere (Karczmarski et al., 2005; Lammers, 2004; Norris et al., 1994; Notarbartolo di Sciara et al., 2009; Oremus et al., 2007; Webster, Cockcroft, & Cadinouche, 2015). Schools were slightly larger than those encountered at Samadai Reef over the same time period (Fumagalli, 2016), but the two populations were similar in including calves and pregnant females in the summer months (Cesario, 2017; Notarbartolo di Sciara et al., 2009) and long‐term resident individuals with high survival rate (Samadai population: *φ* = 0.99 ± 0.02 *SE*; Cesario, 2017). The regular presence of pregnant females and newborn calves in Satayah Reef is consistent with the summer reproductive peak for the Samadai population (Cesario, 2017; Notarbartolo di Sciara et al., 2009). Moreover, both populations were estimated to include ~150–250 adult individuals (Cesario, 2017). With a regional abundance of 6,961 spinner dolphins (CV = 0.26; Costa, 2015), the Samadai and Satayah populations appear to constitute two small components of a much larger community. The literature on the spinner dolphin suggests that resting habitat availability and distribution affect population structure. Specifically, multiple suitable habitats for daily resting support fluid societies, and resting habitats separated by large stretches of pelagic waters are typically inhabited by closed, stable societies (Karczmarski et al., 2005; Norris et al., 1994). As well as geographic isolation, social and ecological factors can also have an influence in shaping the structure of insular communities (Oremus et al., 2007). As it cannot be excluded that these Egyptian units connect to each other and/or to larger, pelagic populations outside the resting areas, the information available suggests that they could be part of a metapopulation, a structure organized in subpopulations of individuals differentially using a network of habitat patches (Levins, 1969).

The population structure is instrumental in assessing the intrinsic vulnerability of the Satayah population. The exposure of calves and resident individuals to interactions with swimmers and boats, the proven sensitivity (Fumagalli et al., 2018), and individual long‐term residence in the study site suggest that this is a vulnerable population, which should be closely monitored to document the occurrence of biological impacts that could be caused, or exacerbated, by the intense WW activities. Such impacts could manifest themselves in two major ways. Firstly, individuals in populations chronically affected by tourism operations and unable to cope with the disturbances may abandon the site and relocate to a less disturbed one (Lusseau, 2004). This is a viable option if alternative suitable sites are available, and the benefits associated with the displacement overcome its risks and costs (e.g., predation, presence of competitors, relations with associates; Frid & Dill, 2002; Gill, Norris, & Sutherland, 2001). However, when this strategy is not advantageous, individuals or groups would continue to use the site despite the disturbances. This can result in changes in demographic parameters, most likely female reproductive success (Christiansen & Lusseau, 2014), and eventually in decreased population size (Bejder et al., 2006; Lusseau, Slooten, & Currey, 2006). A decline in population abundance was reported from Hawaii, where human interactions with spinner dolphins have intensified over the last few decades (Tyne et al., 2017). It is still not clear whether the Egyptian populations are affected by WW interactions, and whether the impacts would lead to displacement or population decline. Furthermore, it must be acknowledged that other phenomena, both natural and anthropogenic (e.g., environmental conditions, resource competition, prey abundance, diseases, overfishing, bycatch) may co‐occur, and their effects interact in threatening wild populations. Although, in most cases, it is extremely complex to tear apart the specific effects of single threats, we recommend future studies to maintain a holistic approach and to quantify, describe, and consider all possible sources of disturbance and stress when assessing the status of the Satayah population. As the 2006 study by Bejder and colleagues demonstrates, control–impact studies would be ideal and should be taken into consideration, when possible. Contrasting and comparing resting behavior within and between control and impact resting areas has already advanced the understanding on the short‐term effects of disturbances on spinner dolphins in Egypt (Fumagalli et al., 2018). As several resting areas are available to spinner dolphins in Egypt and the Red Sea (Fumagalli, Cesario, & Costa, 2019), efforts should be made to (a) compile photoID databases and estimate parameters of the Egyptian populations found at these resting areas, especially those with a potential to be control sites, and to monitor the composition of pelagic schools; (b) quantify and describe the characteristics of WW activities at resting sites, in order to monitor the evolution of the industry and enable the identification of WW variables that may be used in models to measure or predict changes in population demographic parameters (e.g., number of vessels, Bejder et al., 2006; Pérez‐Jorge et al., 2016; implementation of regulations, Gormley et al., 2012); and (c) model individual and population temporal and spatial variation in exposure to anthropogenic stressors (e.g., Pirotta et al., 2015).

In this case study, the duration and characteristics of the study did not allow the assessment of population‐level impacts. Nonetheless, three major direct management applications derive from the investigation of the Satayah population ecology. Firstly, the Satayah population is proposed as a management unit. Current knowledge indicates that the Egyptian spinner dolphins are organized in small, discrete units, whose boundaries are still not understood. If the region hosts a metapopulation, adequate site‐specific management interventions are required to ensure the viability of each subpopulation (Oremus et al., 2007). Secondly, school demographic composition, individual site fidelity, population size, and survival are suggested as key monitoring indicators. Baseline data are now available for future assessment of impacts and resilience of the population, as well as for inclusion in monitoring frameworks (Higham, Bejder, & Lusseau, 2009). Thirdly, the Satayah population is confirmed ideal for the investigation of population trends, as the conditions that maximize power detection—small, resident, easily accessed population, abundance estimates with good precision (Taylor et al., 2007)—are met. However, careful survey planning and design are required to ensure the best compromise between research effort, objectives, and logistical feasibility for prompt detection of changes. At this scope, simulating scenarios of population change and trend detection could help identify such compromise (e.g., Thompson et al., 2000; Tyne et al., 2016). As the current levels of monitoring are inadequate to detect a population decline in a timely manner, and the local WW industry is on the rise and still unregulated, we strongly support recommendations already made by Tyne and colleagues for the spinner dolphins of Hawaii (Tyne et al., 2016). We urge the use of a more cautious approach to the management of such industry, including the reliance on a lower power level (80%) and the adoption of precautionary measures to mitigate impacts to, hopefully, help prevent population decline (Tyne et al., 2016).

We caution here that our findings could have reflected seasonal patterns and, given the high proportion of males in the sample, have over‐represented sex‐specific patterns in residence, which were found at Samadai Reef (Cesario, 2017). Regular surveys throughout the year and a broader temporal and geographic photo‐identification effort are required to further resolve the characteristics of Satayah schools, individual residence and dispersal, to compare trends between study sites, and to advance the understanding of the species organization in the region. In future surveys, adjusting the data collection to apply the Robust Design formulation (Kendall, Pollock, & Brownie, 1995; Pollock, 1982), that best accommodates transience and allows estimation of temporary immigration and emigration, is strongly recommended. Given the preliminary evidence of connectivity between the Samadai and Satayah populations, the opportunity to apply Multistate Robust Design models (Kendall & Bjorkland, 2001; Kendall, Nichols, & Hines, 1997; Pollock, 1982; Schwarz & Stobo, 1997) should also be taken into consideration. Finally, a dedicated survey on the efficacy, precision, implications for capture‐recapture analyses and impacts of surface and underwater photoID data collection is required to define whether one, or a combination of the two, provides the best compromise between research needs and dolphin disturbance.

## 5 MANAGEMENT IMPLICATIONS

This study advanced previous knowledge of the potential disruptive nature of WW on this population (Fumagalli et al., 2018) by showing that operations target‐sensitive segments in a habitat regularly used as a resting and calving ground. We strongly recommend intervention to mitigate disturbance of the population by (a) reducing interactions and exposure rate at Satayah and other resting sites with a time–area closure plan, similar to the one successfully implemented at Samadai Reef, that would best suit spatially and temporally constrained populations (Lusseau, 2014); (b) supporting further research to test the patterns in our results and to monitor population‐level impacts; and (c) devising ways to integrate site‐specific management efforts in a fully developed regional network for the protection of the species. Our experience shows that the assessment of a population vulnerability to WW can greatly benefit from a combination of original, simple, site‐specific information and the pertinent literature. As WW is projected to expand to new territories and populations, this sets an example for other studies in similar contexts.

## CONFLICT OF INTEREST

None declared.

## AUTHORS' CONTRIBUTION

GNS, MC, and MF conceived the idea and designed methodology; MF, AC, and MC collected the data; MF and AC analyzed the data; ES and JH advised on analyses; MF led the writing of the manuscript. All authors contributed critically to the drafts and gave final approval for publication.

## Figures and Tables

**Figure A1:**
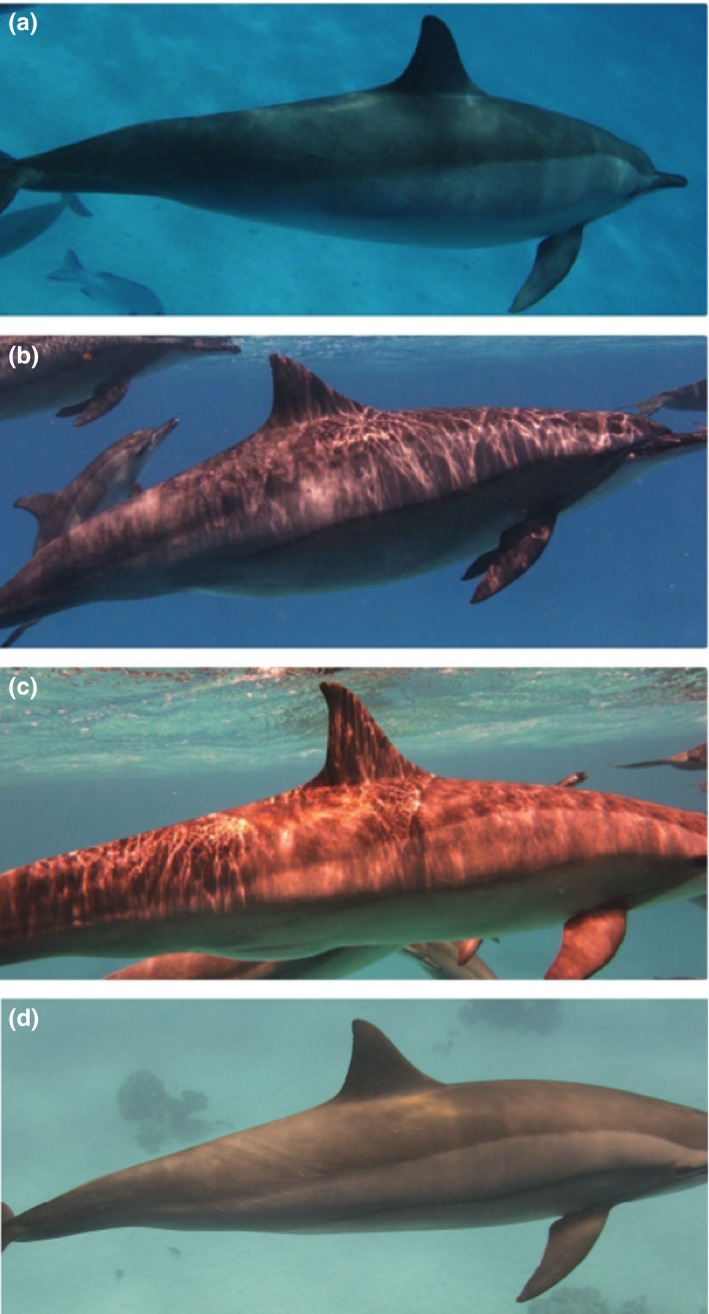
Pregnancy stages of females (modified and adapted from Cesario, 2017)

**Figure A2:**
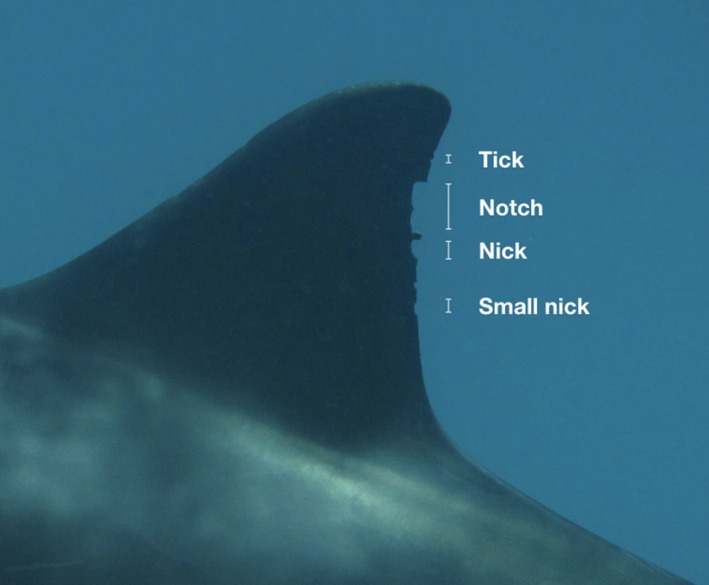
Individual distinctiveness: example of notch, nick, small nick, and tick

## Data Availability

Data available on MF Open Science Framework account at https://osf.io/8w5um/

## Appendix 1

### SCHOOL COMPOSITION

Pregnancy stages of females (Figure A1)

1. Early pregnancy: slightly swollen; approximately midterm pregnancy
2. Late pregnancy: visibly swollen: starting from ca. eight months
3. Postpartum: distended mammaries; for ~2–4 weeks after delivery, recognizable regardless of the association with a neonate
4. Nonpregnant female

### PHOTOGRAPHIC IDENTIFICATION

#### Code of conduct for photographic sessions

##### Underwater

The code of conduct prescribed quiet and gentle snorkeling to the side of the group, without arm movements, splashes, noises, or direct, abrupt, frontal approaches. Researcher attempted to approach the group to an ideal distance <5 m and to collect pictures at depth >50 cm to avoid sunlight reflection on the profile of the dorsal fin.

##### Surface

Speedboat approaches were carried out at constant low speed, avoiding abrupt changes of direction and gears, and always to the side of the dolphin group.

##### Equipment

Equipment for underwater sessions Lumix TZ‐7, Canon PowerShot S110 and D10; for surface session Canon 500D and 7D with 70–200 mm lenses.

#### Photographic quality and distinctiveness

Each image was scored according to four photographic quality criteria (focus, contrast, angle, and fin visibility) adapted from Friday et al. (2000) and Urian et al. (2015), and consistent with Samadai photo‐identification studies (Cesario, 2017) (Table A1). The sum of the criteria scores defined excellent, very good, good, fair, and poor photographic quality categories (Table A2).

**Table A1:** List of criteria scores used for the assessment of photographic quality

Criteria	Ideal	Good	Moderate	Poor
Focus/clarity	1	2	4	9
Contrast	1	–	–	3
Angle	1	–	2	8
Fin visibility	0	–	2	8

**Table A2:** Categories of photographic quality and the corresponding scores

Photo quality	Sum of scores
Excellent	3–4
Very good	5–6
Good	7–8
Fair	9
Poor	11+

Each dorsal fin displayed in Excellent and Very Good images was assessed for individual distinctiveness. The number of notches (large marks, ca. 1/6 of the fin profile), nicks (medium marks, ca. 1/18), small nicks (small marks, <1/18), and ticks (minor indentations) on the dorsal fin (Figure A2) were used to categorize individuals as “very distinctive” (D1), “distinctive” (D2), “marked” (D3), and “not marked” (D4, D5). D1 and D2 together are Highly Marked Individuals (HMIs).

### POPULATION PARAMETERS

#### Capture–Recapture (CR) assumptions

The CR models employed assume that marks do not affect the behavior or fate of individuals (*trap response*) and are not lost, misread, overlooked, or missed (*mark loss*); every individual alive at time *i* has the same probability of capture (*equal catchability*); the fate of each marked individual is independent of the fate of other marked individuals (*independence of fates*); no birth, death, immigration, and emigration occur during the resampling process (*instantaneous sampling*) (Lindberg & Rexstad, 2006). When needed, these assumptions can be relaxed, accommodated, or corrected (White & Burnham, 1999). To enhance validation, we adopted the methods and strategies described in Table A3.

**Table A3:** Capture–recapture assumptions, definition from Lindberg & Rexstad (2006), diagnostic tools, and strategies to enhance validation employed in this study

Assumption	Description	Test	Validation
Trap response	Marks do not affect the behavior or fate of the marked individuals	Pradel's test for trap dependence	Survey design: Photo‐identification does not require capture, handling, or physical marking, thus unlikely to cause stress and behavioral response (Pollock et al., 1990; Pradel, 1993; Williams, Trites, & Bain, 2002)
Mark loss and recognition	Marks are not lost, missed, overlooked or misread		Data processing: Highly marked individuals only; High‐quality pictures (Barlow et al., 2011; Frasier, Hamilton, Brown, Kraus, & White, 2009); Experienced cataloguer (Pollock et al., 1990; Williams, Nichols, et al., 2002)
Equal catchability	Every marked individual alive in the population at time *i* has the same probability of capture	Pooled chi‐squared statistics (Test 2 + Test 3)	Survey design: Area surveyed correspond with home range; Seasonal phenomena that may affect individuals’ presence are taken into consideration (Hines, Kendall, & Nichols, 2003). Data collection: Even coverage of groups
Independence of fates	The fate of each marked individual is independent of the fate of other marked individuals		Data processing: Exclude individuals not mixing at random (e.g., calves) (Rosel et al., 2011)
Instantaneous sampling	Resampling is instantaneous; that is, birth, death, immigration, and emigration do not occur during the resampling process		Survey design: Sampling occasions are short in duration (Pollock et al., 1990; Williams, Nichols, et al., 2002)

#### FORMULAE

##### Horvitz–Thompson type estimator

HMI = Highly Marked Individuals; *N*_HMIi_ = estimated number of Highly Marked Individuals at occasion *i*; *SE*_NHMIi_ = standard error of *N*_HMIi_; 95CI_NHMIi_ = 95% confidence interval of *N*_HMIi_; *n*_HMIi_ = number of Highly Marked Individuals captured at occasion *i*; *p_i_* = capture probability at occasion *i*; var(pi) = variance of capture probability at occasion *i*(Loery, Nichols, & Hines, 1997; McDonald & Amstrup, 2001).

$$N_{\text{HMIi}}=\frac{n_{\text{HMIi}}}{p_{i}}$$

$$SE_{\text{NHMIi}}=\sqrt{\frac{\text{var}\left(p_{i}\right)}{p_{i}^{2}}{\left(\frac{n_{\text{HMIi}}}{p_{i}}\right)}^{2}}$$

$$95CI_{\text{NHMIi}}=N_{\text{HMIi}}\pm 1.96\;SE_{\text{HMIi}}$$

##### Mark rate

*θ* = Mark Rate, *SE_θ_* = standard error of *θ*

$$\theta =\frac{\text{number}\;\text{of}\;\text{highly}\;\text{marked}}{\text{total}\;\text{number}\;\text{of}\;\text{individuals}}$$

$$SE_{\theta }=\sqrt{\frac{\theta \left(1-\theta \right)}{\text{sample}\;\text{size}}}$$

##### Total population size

*N* = estimate of the total population size; *SE_N_* = standard error of *N*; 95CI*_N_* = 95% confidence interval of *N* (Burnham et al., 1987; Williams, Nichols, et al., 2002).

$$N=\frac{N_{\text{HMI}}}{\theta }$$

$$SE_{N}=\sqrt{N^{2}\frac{SE_{\text{HMIi}}^{2}}{N_{\text{HMIi}}^{2}}+\frac{1-\theta }{n\theta }}$$

$$C=\exp\left\{1.96\sqrt{\ln\left[1+{\left(\frac{SE_{N}}{N}\right)}^{2}\right]}\right\}$$

$$95{\text{CI}}_{N}=\frac{N}{C}÷NC$$

### POWER ANALYSES FOR POPULATION TRENDS

*A_i_* = abundance at occasion *i*; *A*_1_ = initial abundance; CV = coefficient of variation; *n* = the number of samples; *r* = fractional rate of change of the quantity being measured; *α* and *β*, the probabilities of Type 1 and 2 errors, $\sigma_{\text{res}}^{2}$ = estimate of residual variance (Gerrodette, 1987).

$$A_{i}=A_{1}{\left(1+r\right)}^{i-1}$$

$$r^{2}n^{3}\geq 12{\text{CV}}^{2}{\left(z_{\frac{\alpha }{2}}+z_{\beta }\right)}^{2}$$

$$r={\left(R+1\right)}^{\frac{1}{n-1}}-1$$

$$SE_{r}=\sqrt{\frac{12\sigma_{\text{res}}^{2}}{n\left(n+1\right)\left(n-1\right)}}$$

$$\sigma_{\text{res}}^{2}={\left({\text{CVA}}_{1}\right)}^{2}\left\{1+r\left(n-1\right)\left[1+\frac{r}{6}\left(2n-1\right)\right]\right\}.$$
